# Docking Studies and Molecular Dynamics Simulations of Potential Inhibitors from the Brown Seaweed *Sargassum polycystum* (Phaeophyceae) against PLpro of SARS-CoV-2

**DOI:** 10.3390/biotech12020046

**Published:** 2023-06-11

**Authors:** Asmi Citra Malina A. R. Tassakka, Israini Wiyulanda Iskandar, Jamaluddin Fitrah Alam, Andi Dian Permana, Muhammad Nasrum Massi, Sulfahri, Jamaluddin Jompa, Lawrence Manzano Liao

**Affiliations:** 1 Faculty of Marine Science and Fisheries, Universitas Hasanuddin, Makassar 90245, Indonesia; alamjamaluddin@unhas.ac.id (J.F.A.); j.jompa@unhas.ac.id (J.J.); 2 Biomedicine, Graduate School, Universitas Hasanuddin, Makassar 90245, Indonesia; israiniwiyulanda@gmail.com; 3 Faculty of Pharmacy, Universitas Hasanuddin, Makassar 90245, Indonesia; andi.dian.permana@farmasi.unhas.ac.id; 4 Faculty of Medicine, Universitas Hasanuddin, Makassar 90245, Indonesia; nasrumm2000@yahoo.com; 5 Faculty of Mathematics and Natural Sciences, Universitas Hasanuddin, Makassar 90245, Indonesia; sulfahri@unhas.ac.id; 6 Graduate School of Integrated Sciences for Life, Hiroshima University, Higashi-Hiroshima 739-8528, Japan; lliao@hiroshima-u.ac.jp

**Keywords:** algae, computer modelling, molecular docking, natural compounds, Phaeophyceae, virus

## Abstract

The COVID-19 disease is a major problem affecting human health all over the world. Consequently, researchers have been trying to find solutions to treat this pandemic-scale disease. Even if there are vaccines and approved drugs that could decrease the spread of this pandemic, multidisciplinary approaches are still needed to identify new small molecules as alternatives to combat COVID-19, especially those from nature. In this study, we employed computational approaches by screening 17 natural compounds from the tropical brown seaweed *Sargassum polycystum* known to have anti-viral properties that benefit human health. This study assessed some seaweed natural products that are bound to the PLpro of SARS-CoV-2. By employing pharmacophore and molecular docking, these natural compounds from *S. polycystum* showed remarkable scores for protein targets with competitive scores compared to X-ray crystallography ligands and well-known antiviral compounds. This study provides insightful information for advanced study and further in vitro examination and clinical investigation for drug development prospects of abundant yet underexploited tropical seaweeds.

## 1. Introduction

Severe Acute Respiratory Syndrome-Coronavirus-2 or SARS-CoV-2 has become a transmissible pandemic virus targeting the respiratory system that rapidly and seriously affects global health, causing the coronavirus disease in 2019 (COVID-19) [1]. This disease was initially discovered in Wuhan City, China, in December 2019 and has now spread to approximately 230 countries [2]. Several symptoms are found in patients infected by COVID-19, mainly including dry cough, body ache, high fever, fatigue, difficulty breathing, pressure build-up in the chest cavity, and speech difficulties [3]. Specifically, the endothelial cells of the lung are the main targets where the SARS-CoV-2 binds. This would then stimulate an immune response and causes cytokine storm syndrome [4,5]. Afterward, this results in fatal respiratory failure, which is considered the primary reason for mortality in COVID-19 patients. Furthermore, about 6.34 million deaths have been reported until the mid-year of 2022, and an average of 3.39 million new cases weekly in June 2022 have been registered, despite a total of 12,037,259,035 vaccine doses being administered. The World Health Organization (WHO) has therefore acknowledged COVID-19 as a worldwide pandemic in this century [2,6,7]. 

Despite its lethality, no selective treatment using antiviral drugs to treat COVID-19 exists [8]. Instead, the patients are mainly administered supportive treatment. In an attempt to overcome this issue, more than 200 drugs which FDA has previously approved have been used for clinical experiments [9]. Numerous antiviral agents, namely ritonavir, lopinavir, remdesivir, favipiravir, and oseltamivir, as well as antimicrobial agents, namely antiprotozoals and azithromycin, and anti-inflammatory drugs, including glucocorticoids, hydroxychloroquine, and chloroquine, have been tried for COVID-19 management and treatment. Nevertheless, the administration of these drugs could potentially result in high toxicity among treated patients [10,11]. Accordingly, novel treatment using new compounds to target the virus directly is immediately required to control the COVID-19 pandemic.

Among several resources, the use of natural medicines from medicinal plants, which have been reported to possess a rich and wide range of bioactive compounds, has been extensively investigated in treating various diseases. Currently, the discovery of bioactives from marine sources has attracted the interest of researchers worldwide. For example, ascidians have been reported to produce secondary metabolites with antimicrobial properties. Additionally, in 2021, a total of 1425 new compounds have been identified as bioactive substances [12,13]. Moreover, to address the eradication of COVID-19, the application of bioactive compounds from medicinal plants has been considered an alternative treatment [14,15] as they have been found to be highly effective with less toxicity. In the field of drug discovery, the application of computational techniques has become the main option, especially in addressing urgent requirements for new drugs. Compared to other approaches, the computational approach does not entail high costs and a long time. Essentially, numerous studies have reported its successful application in screening for potential benefits of several natural products as bioactive agents to target SARS-CoV-2 [6,11,16,17]. Specifically, we have previously reported the potential antiviral activity of the marine red alga *Halymenia durvillei* Bory in inhibiting SARS-CoV-2 using a computational study. Our study found that the bioactive compound in the red alga showed a high affinity to 3CL-Mpro, which is an essential enzyme for SARS-CoV-2 replication [18]. Furthermore, in addition to 3CL-Mpro, another protein, namely PLpro, has also been reported to play essential roles in the replication of SARS-CoV-2.

In this study, we investigated the potential antiviral activity of another type of seaweed, the marine brown alga *Sargassum polycystum* C. Agardh using an in silico computational model against SARS-CoV-2. This plant has been discovered to show antiviral activity against numerous virus-associated diseases. For the first time, we investigate the bioactive compounds from *S. polycystum* against PLpro using an in-silico approach. Ethanolic compounds further analyzed using GC-MS were screened for their antiviral activities. This study provides new information about the potential anti-SARS-CoV-2 agents from *S. polycystum* and reinforces previous findings by applying the computational approach as an initial step to discovering new COVID-19 treatments.

## 2. Materials and Methods

### 2.1. Sample Collection and Extraction

Samples of the brown seaweed *Sargassum polycystum* were collected from around Lae-Lae Island, Makassar, Indonesia (119°20.52′ E, 5°7.38′ N). The samples were identified by the use of morphometric techniques. The Faculty of Marine Science and Fisheries at Hasanuddin University is where voucher samples are kept. Algae samples were dried in a herb drier after being cleaned with distilled water to eliminate any remaining particles. Dried algae (90.8 g) were macerated after adding 700 mL of 96% ethanol. Then, the mixture was filtered using a rotary evaporator.

### 2.2. Analysis of Extracts Using Gas Chromatography-Mass Spectrometry (GC-MS)

The bioactive elements of *Sargassum polycystum* extracts were discovered using an Ultra Shimadzu QP2010 Gas Chromatograph Mass Spectrometer paired with an AOC-20i Autosampler. An SH-Rxi-5Sil capillary column MS with a 30 m column length and 0.25 mm inner diameter was utilized for the analysis, and a Vigreux column with a 20 cm length and 2.4 cm inner diameter and an injection volume of 1 L was employed for vacuum fractionation distillation. A 250 mL round bottom flask was filled with 5 g of *S. polycystum* extract. The flask was next attached to a vacuum pump, fractionation column, and batch jacket, which heated it to 200 °C under 96 kPa of pressure. The distillates were collected at each change in steam temperature during the distillation procedure, and the amounts of alcohol in each fraction were measured. For example, helium carrier gas was used in GC-MS analysis with pressure at 76.9 kPa, injector temperature in splitless mode at 250 °C, carrier gas flow rate at 14 mL/min, and a ratio of 1:10. The ion source and interfaces were 200 °C and 280 °C, respectively, in temperature. The solvent cut-off time was 3 min, and the mass spectrum ranged from 400 to 700 *m*/*z*. With a hold time of two minutes, the column’s starting temperature was 110 °C. With a final temperature of 280 °C and a holding period of 9 min at a rate of 5 °C/min, the analysis was carried out at a rate of 10 °C/min up to 200 °C. It took 36 min to complete. The NIST and Wiley libraries were used to determine the bioactive chemicals [19].

### 2.3. Preparation of Ligands and SARS-CoV-2 PLpro

The Chimera and PyMoL were used to prepare the protein structures and the ligands. SARS-CoV-2 enzyme structure was obtained from the Protein Data Bank (PDB) for protein preparation: PLpro (7JIR) with resolution 2.79 Å. Then, the protein structure was prepared for the docking approach by removing water and non-standard molecules, adding hydrogen, extracting X-ray crystallography ligand (Snyder 457), adding charged ions, and doing minimization with Gasteiger force field in UCSF Chimera. Meanwhile, all of the three-dimensional structures and canonical smiles from GC/MS data, as well as inhibitors, were gathered in ligand preparations from PubChem (https://pubchem.ncbi.nlm.nih.gov/, accessed on 23 March 2022). Any compounds were generated using UCSF Chimera 1.14 (https://www.cgl.ucsf.edu/chimera/, accessed on 23 March 2022) by adding hydrogens and charges, and all unavailable 3D compound structures were converted using the NIH molecule converter at https://cactus.nci.nih.gov (accessed on 25 March 2022). The compounds were then prepared into their 3D structure using the Gasteiger Force Field in UCSF Chimera and then further screened in the pharmacophore and molecular docking study. 

### 2.4. Pharmacophore-Based Virtual Screening

The pharmacophore study used LigandScout version 4.4 to analyze pharmacophore interaction features of protein and ligand structures. The structure-based pharmacophore model was initiated by creating a pharmacophore model from the initial ligand of the co-crystallized protein structure Snyder 457 from PLpro (7JIR PDB). Thereafter, the hit compounds from virtual screening are analyzed for their pharmacophore features.

### 2.5. Molecular Docking

The Scripps Research Institute’s AutoDock Tools version 1.5.6 and Cambridge Crystallographic Data Centre’s GOLD were both used for the docking study. A dataset of 17 natural compounds from *S. polycystum* alga and the co-crystallized ligand (Snyder 457) were used for molecular docking. Grid size is manually defined according to the active site of the X-ray crystallography PLpro structure, and the exhaustiveness were set by default. The grid scale is 50.23, 29.9, 0.74 (*x*, *y*, *z*), and the box size is 20 × 20 × 20. Moreover, the docking results were analyzed based on a combination of binding energy, ligand conformations, binding site, and favorable interactions. The best binding affinity and fitness scores from compounds were analyzed for their molecular interactions with active site residues required for the biological activity of the virus.

### 2.6. Docking Validation and Ligand Efficiency

In validating the docking results, the re-docking method of the co-crystalized ligand, Snyder 457, was applied as the positive control. Using AutoDock, the ligand was taken out of the protein’s active site and docked back at the original location. The docked co-crystallized was then superimposed with the re-docked protein-ligand complex result, and the root mean square deviation was calculated using PyMOL. The docking result was accepted if the docking co-crystallized ligand and the re-docked co-crystallized ligand bound to the protein have the same position at the active site with a similar pose mode and low rmsd. These steps were performed to validate the docking procedure to ensure the validation of the docking [20]. Each ligand’s efficiency should also be determined in accordance with its affinity and size since this will help a chemical be further optimized. The quantity of non-hydrogen atoms in the ligand that were taken into consideration affects the −ΔG or free energy of binding of each ligand to HA [21].

### 2.7. Molecular Dynamics

GROMACS 2021.5 was used for MD simulations for the PLpro-Cholesterol and PLpro-Snyder 457 complex for 50 ns. The unit cell—a dodecahedron box with a minimum separation from the protein surface of 1 nm—was solvated using the Simple Point Charge (SPC) water model. The topologies of the target were constructed using the CHARMM36 force field. The system was subjected to energy minimization before the MD run using the steepest descent integrator for 50,000 steps with a force convergence of 1000 kcal/mol/nm. After that, each protein-ligand combination was equilibrated using NVT and NPT ensembles within 100 ps. The systems were connected to temperature and pressure controllers by Berendsen and Parrinello-Rahman, respectively, to maintain a temperature of 300 K and a pressure of 1 bar throughout equilibration. MD simulations were run for 50 ns, and the system’s coordinates were stored every 2 fs. The duration of 50 ns is considered a sufficient time to achieve a balance between computational time and information. When observing the fluctuation graph, the data provided already show a stable equilibration. Several analytic modules included in the GROMACS package were used to undertake structural and conformational analysis of all systems. The snapshots’ binding free energies were treated to MM-PBSA (MMPoisson-Boltzmann surface area).

## 3. Results

### 3.1. GC/MS

**Figure 1:**
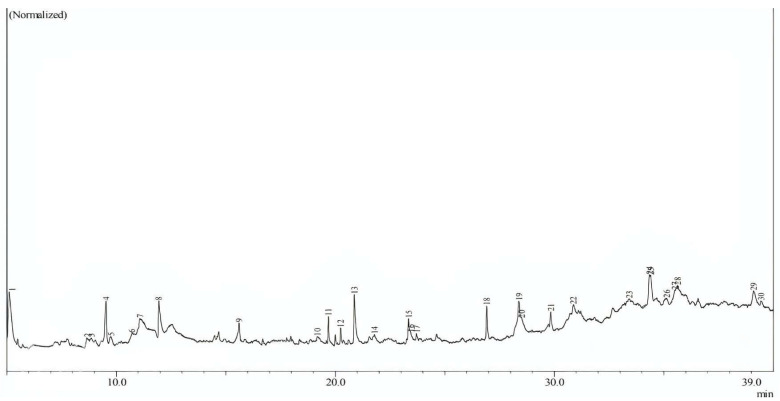
Chromatogram GC/MS of the extraction of natural compounds *Sargassum polycystum*.

**Figure 2:**
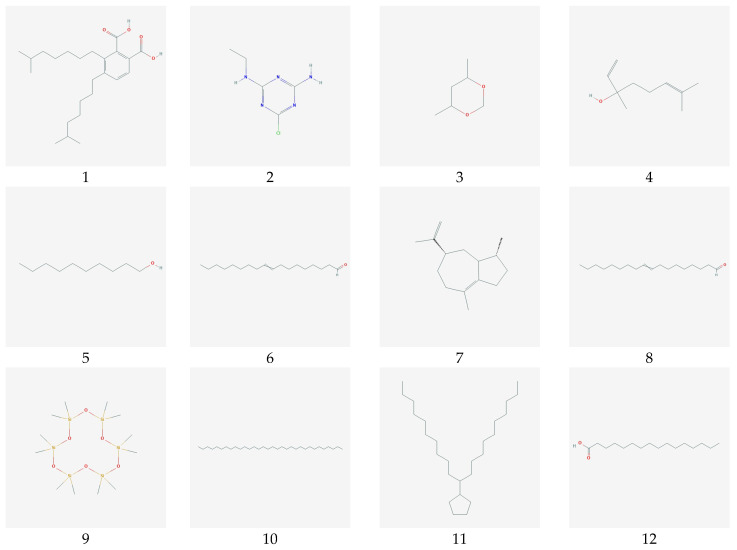

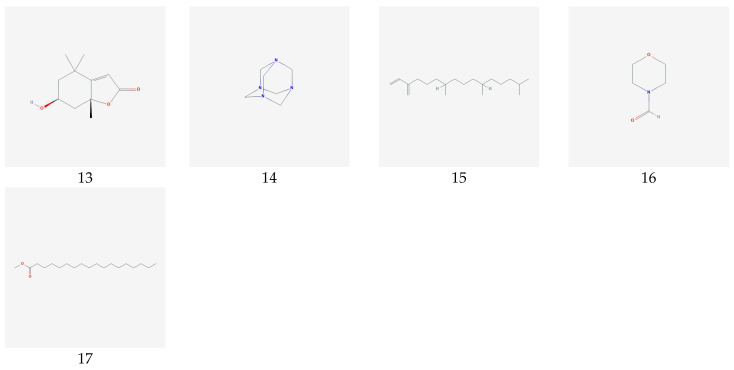
The chemical structure 17 natural compounds *Sargassum polycystum*.

In this study, phytochemicals from *Sargassum polycystum* were extracted using ethanol as the solvent, resulting in the identification of 17 compounds. The GC–MS chromatograms of these compounds are presented in Figure 1, and detailed information about each compound can be found in Table 1. Analysis of Table 1 reveals that the compound with the highest area percentage (12.85%) is 1,3,5-triazine-2,4-diamine,6-chloro-n-ethyl (C2). On the other hand, the compound with the lowest area percentage (0.99%) is Cyclohexasiloxane, Dodecamethyl (C9). Following these, Methenamine (C14) exhibits an area percentage of 6.73%, while Hexadecanoic Acid (C12) and 1,3-dioxane,4,6-dimethyl (C3) show area percentages of 6.66%, respectively. Other compounds display areas of less than 5%. The majority of the identified compounds are fatty acids or lipids, constituting the prominent phytochemicals derived from this seaweed, as shown in Table 1 (refer to Figure 2).

### 3.2. Identification of 3D-Pharmacophore

**Figure 3:**
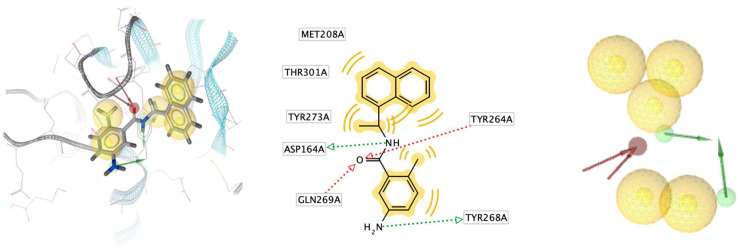
Structure-based pharmacophore model from PLpro enzyme with its inhibitor Synder 457.

**Figure 4:**
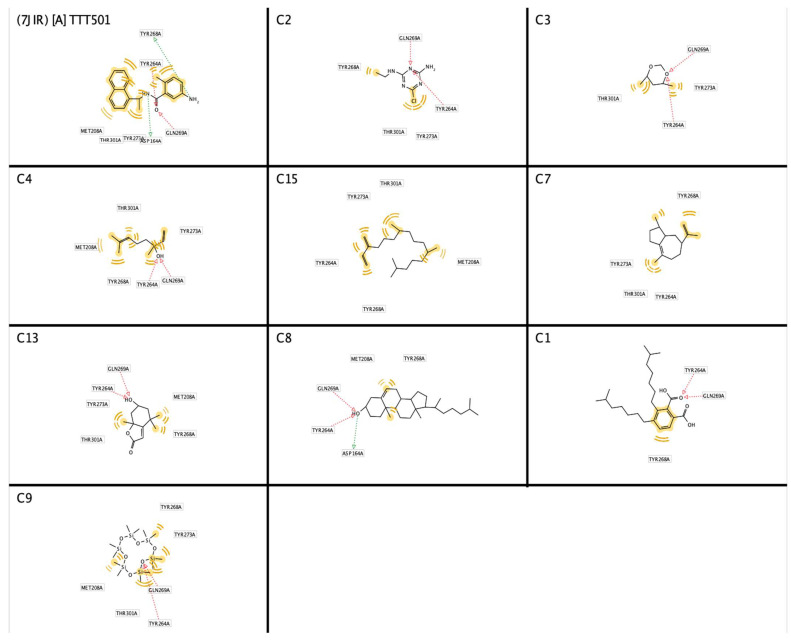
Structure-based pharmacophore screening result of PLpro inhibitors (red arrows, hydrogen bond acceptor (HBA); greens arrow, hydrogen bond donor (HBD); yellow lines, hydrophobic sites). A. Snyder 457 (2HBA;2HBD), C1(2HBA;0HBD), C2(2HBA;0HBD), C3(2HBA;0HBD), C4(2HBA;0HBD), C7(0HBA;0HBD), C8(2HBA;1HBD), C9(2HBA;0HBD), C13(2HBA;0HBD), and C15(0HBA;0HBD).

The identification of a structure-based pharmacophore model from PLpro enzyme with its inhibitor Snyder 457 (see Figure 3) revealed five hydrophobic interactions with Met208, Tyr264, Tyr268, Gln269, Tyr273, and Thr301, two hydrogen-bond acceptors from Gln269 and Tyr264 with O atom and two hydrogen-bond donors from Asp164 and Tyr268 with NH and NH_2_, respectively. Furthermore, the virtual screening result from the pharmacophore model of Snyder 457 yielded nine natural compounds of *S. polycystum* as follows: C1, C2, C3, C4, C7, C8, C9, C13, and C15 (see Figure 4). The highest pharmacophore-fit score was C4 with four hydrophobic interactions and a 66.04 score with two hydrogen bond acceptors at the OH atom.

### 3.3. SARS-CoV-2 Enzyme Docking PLpro

To gain insight into the molecular interaction of PLpro enzyme or papain-like protease enzyme that regulates SARS-CoV-2 virus replication, 17 natural compounds of *Sargassum polycystum* have been screened in molecular docking. Through assessing compounds, the most potent compounds have been examined based on their binding affinity and molecular interaction. The most potential compounds based on AutoDock calculation (see Table 2) were C8 and C7 with −6.5 kcal/mol, which was lower than the positive control Snyder 457 with −10 kcal/mol. The compounds were then followed by C1 (−6.0 kcal/mol), C6 (−5.5 kcal/mol), and C12 (−5.4 kcal/mol). Meanwhile, based on GOLD scoring (see Table 3), the best natural compound was C11 with 39.08 which was slightly lower than Snyder 457 with 42.04, and the score of C11 was higher than C8 to around 10 scores.

**Figure 5:**
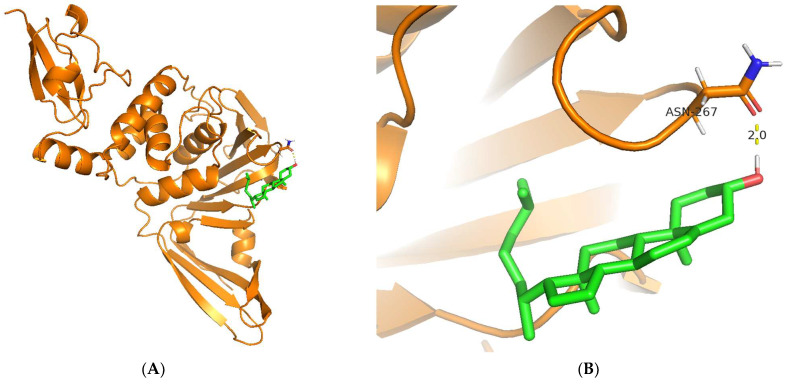

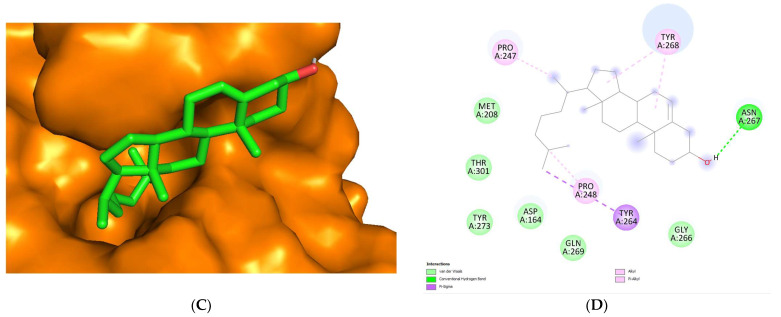
Cartoon representation of Cholesterol (green) bound to the active site of PLpro enzyme (orange) (**A**,**B**). Hydrogen-bond and other molecular interactions between Cholesterol and the active residue of PLpro (**B**,**D**). Surface representation of Cholesterol bound to the active site of PLpro (**C**).

**Figure 6:**
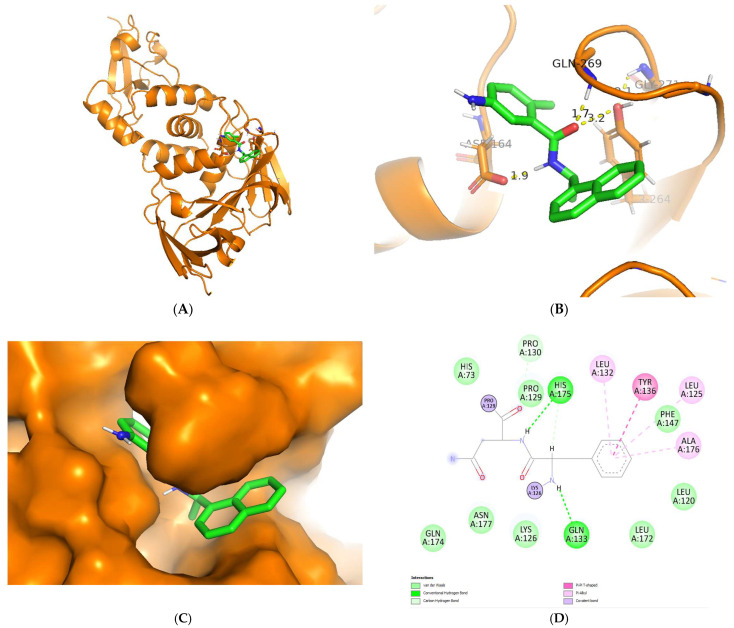
Cartoon representation of Snyder 457 (green) bound to the active site of PLpro enzyme (orange) (**A**,**B**). Hydrogen-bond and other molecular interactions between Snyder 457 and the active residue of PLpro (**B**,**D**). Surface representation of Snyder 457 bound to the active site of PLpro (**C**).

Regarding the visualization of molecular docking (see Figure 5), the conformation of C8 bound to the cleft of the enzyme was not deep enough, such as Snyder 457 (see Figure 6), but both compounds were fairly attached to the active site of the enzyme. The interaction between ligand and protein contained several residues with 5 Å at the active site: Cys111, Lys157, Leu162, Gly163, Asp164, Val165, Arg167, Ala246, Pro247, Pro248, Tyr264, Gly266, Asn267, Tyr268, Gln269, Gly271, His272, Tyr273, and Thr301. The compounds of C8 formed one hydrogen bond with Asn267 and developed a Pi-Sigma interaction with Tyr264. Similarly, Snyder 457 formed several hydrogen bonds with PLpro active site residues Asp164, Gln269, and Tyr264, where Asp164 generated Pi-Anion bonding interaction with the ligand.

### 3.4. SARS-CoV-2 Enzyme Molecular Dynamics PLpro

**Figure 7:**
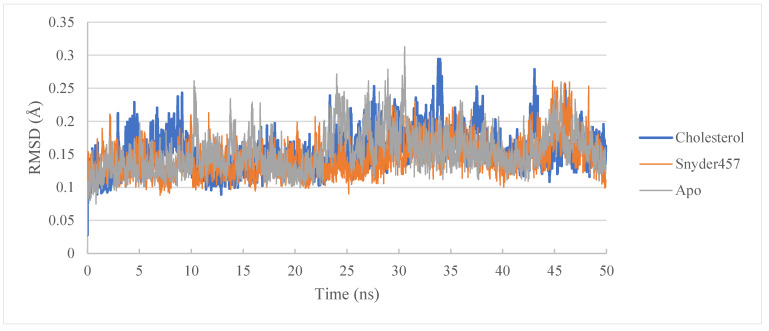
RMSD of PLpro Apo (Grey), PLpro-Cholesterol (Blue), and Plpro-Snyder 457 (Orange).

The molecular dynamics study of PLpro-Cholesterol complex, PLpro-Snyder 457 complex, and the apo system continued to understand further the flexibility degree. Gromacs version 2021.5 was employed to carry out the investigation (see Table 4). Temperature, pressure, and potential/kinetic energy were examined as quality control parameters for the simulated system to ensure that the simulations were accurate. The root means square deviations (RMSD) of the C-alpha atoms of each complex were computed to investigate the complexes’ rigidity (see Figure 7). The average RMSD in the PLpro-Cholesterol complex is 0.15 nm, and the PLpro-Snyder 457 complex is about 0.14 nm. Because of their greater flexibility, the apo PLpro, the complexes of Cholesterol, and Snyder 457 showed a fairly stable fluctuation in RMSD. Around 10 ns, the apo PLpro achieved a stable state, and after 10 ns, the PLpro-Cholesterol and PLpro-Snyder 457 also stabilized. In both systems, there is some variation after 25 ns, but it remains constant for the rest of the simulation duration.

**Figure 8:**
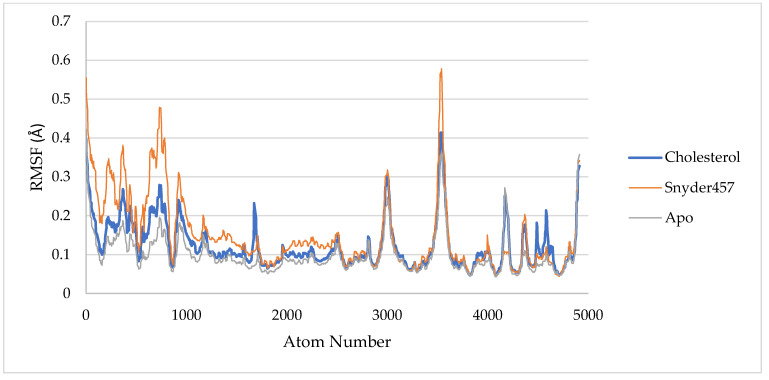
RMSF of PLpro Apo (Grey), PLpro-Cholesterol (Blue), and PLpro-Snyder 457 (Orange).

For estimating residue flexibility during dynamics of the targeted protein, the backbone atoms of each amino acid residue of PLpro in the Apo PLpro, PLpro-cholesterol, and PLpro-Snyder 457 complex are calculated and presented in Figure 8 as RMSF (Root Mean Square Fluctuations). In PLpro-Cholesterol, the average RMSF is about 0.12 nm, and in PLpro-Snyder is slightly higher at 0.15 nm. Otherwise, the average apo system has lower rmsf with 0.1 nm. The spikes of each system can be seen at the same locations as shown in Figure 8 with Thr225, Cys226, Gly227, and Lys228.

**Figure 9:**
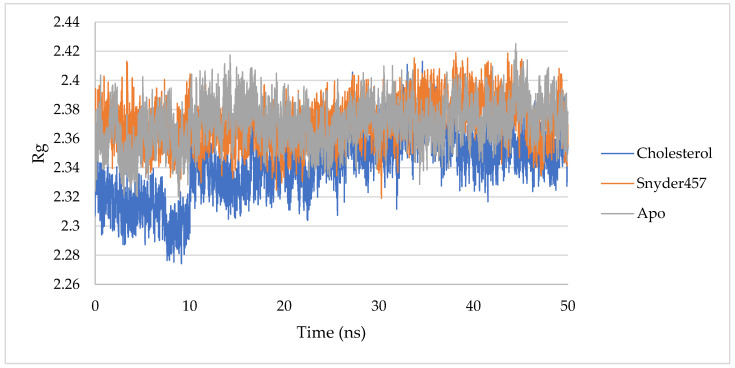
RoG of PLpro Apo (Gray), PLpro-Cholesterol (Blue), and PLpro-Snyder 457 (Orange).

The degree of protein compaction is indicated by the gyrating radius. A collection of atoms’ mass-weighted RMSD is calculated from their shared center of mass. As a consequence, the examination of the Rg’s trajectory shows how the protein’s overall dimension has changed during dynamics. Figure 9 compares the findings of radius of gyration (Rg) studies for the apo PLpro, the PLpro-Snyder 457 complex, and the PLpro-Cholesterol complex. PLpro-average Cholesterol’s Rg value was 2.34 nm, with a considerable decline occurring before 15 ns (Figure 9). On the other hand, the PLpro-average Snyder 457’s Rg value was around 2.37 nm, the same as the Rg of the Apo system.

Binding free energy is the total of all the non-bonded interactions. It was estimated for PLpro-Cholesterol and PLpro-Snyder 457 using the MM-PBSA method. From the results obtained from the MM-PBSA analysis, the average binding free energies (ΔGBind) of PLpro-Cholesterol and PLpro-Snyder 457 complexes were calculated during the 50 ns simulation. The resulting ΔGBind of PLpro-Cholesterol and PLpro-Snyder 457 complexes were found to be −20.91 kJ mol^–1^ and −30.66 kJ mol^–1^, respectively. The details of the MM-PBSA calculation of the complexes are summarized in Table 5.

## 4. Discussion

The utilization of seaweeds has improved and benefitted many aspects of human life. The consumption of seaweeds in Asia, either as part of the traditional diet or therapeutic alternative, appears to be growing in the West. Seaweeds contain many kinds of important natural compounds which have been proven experimentally to prevent or treat some diseases. Seaweed compounds include polysaccharides, minerals, antioxidants, and lots of essential nutrients such as fatty acids, which could promote human health as antiviral, antitumor, anti-inflammatory, and anticancer agents [22]. Recent studies have highlighted the significant nutritional value of seaweeds and the remarkable properties exhibited by their bioactive compounds. Seaweeds belonging to the classes Phaeophyceae, Rhodophyta, and Chlorophyta are known to contain unique compounds that offer various potential health benefits. Research has indicated that the consumption of seaweeds is associated with a reduced risk of ischemic heart disease and lower mortality from stroke, possibly due to their ability to regulate blood pressure and lower lipid levels. These findings emphasize the potential role of seaweeds as valuable allies in promoting our overall health and well-being [23,24].

Although many studies have identified potential seaweed natural compounds for various medicinal applications, the search for natural compounds useful as effective drugs, vaccines, and dietary supplements for the current COVID-19 pandemic remains a high priority for preserving human health. Recent in silico studies have highlighted the health benefits of seaweed, particularly in the context of COVID-19. These studies have revealed the potential of seaweed compounds to inhibit the binding of the omicron B.1.1.529 spike protein’s receptor-binding domain (RBD) with the ACE2 receptor, which plays a crucial role in the viral entry process. Additionally, research on aldehyde derivatives from seaweeds has shown promising results in the fight against SARS-CoV-2. Specifically, 3,4-dihydroxybenzaldehyde has been predicted to exhibit interactions with the 3C-like protease, an important viral enzyme. These findings suggest that seaweed compounds have the potential to be developed as effective therapeutics against COVID-19 by targeting key viral proteins and inhibiting viral replication.

In the current study, the assay of 17 natural compounds from the brown seaweed *Sargassum polycystum* identified promising antagonistic action against the SARS-CoV-2 enzymes PLpro, which play an important role in the metabolism of the SARS-CoV-2. Therefore, their potential as antivirals was investigated by computer-aided drug discovery, which consists of a pharmacophore study (ligand-based pharmacophore and structure-based pharmacophore model), virtual screening, and molecular docking. Among the 17 natural compounds found in *S. polycystum*, fatty acids are common and have shown remarkable results compared to co-crystalized ligands and repurposed drugs against the SARS-CoV-2 enzymes. Intriguingly, some studies reported that fatty acids have antiviral, antibacterial, and antifungal activity. Studies of fatty acids as antiviral agents have shown that they could inhibit viral enzymes either through their replication or expression genes [25]. Furthermore, a recent study of SARS-CoV-2 inhibitors has found that polyunsaturated, monounsaturated, and saturated fatty acids could inhibit the receptor of SARS-CoV-2 entry, hACE2 [26].

PLpro enzyme plays an essential role in processing viral polyproteins to produce a functional replicate complex, reproduction of the virus, and immune system responses. It is one of the SARS-CoV-2 proteases that was targeted for the development of antivirals [27,28]. The structure of PLpro (7JIR PDB) consisted of 318 amino acids, 9 α-helices, 19 β-sheets, and 27 loops, while the active sites of the enzyme were located around the helices α5, α8, 13β-15β, 18β, loop15, and loop22. The inhibitor assay of the crystallized ligand PLpro and the list of natural compounds against the PLpro enzyme were analysed through pharmacophore and molecular docking methods. In structure-based pharmacophore, which is based on pharmacophore features from Snyder 457, it was revealed that 1,6-octadien-3-ol, 3,7-dimethyl is the promising inhibitor for the PLpro enzyme. The pharmacophore feature of Synder457, also known as GRL0617, was used as the model due to its potential to be an inhibitor of PLpro. The ability of Snyder 457 to induce a conformational change of PLpro has been investigated by the molecular dynamics approach [29]. Therefore, this study has conducted the prior step in molecular docking using two standard docking software, AutoDock, and GOLD, to assess and compare the ability of the 17 natural compounds from *S. polycystum* against Snyder 457. Both AutoDock and GOLD yielded different but promising results, in which Cholest-5-en-3-ol (3.Beta.) was identified by AutoDock and Heneicosane, 11-cyclopentyl by GOLD as candidate inhibitors for PLpro. Furthermore, previous in silico studies have provided insights into the potential health benefits of specific compounds found in seaweed. For example, Cholesterol, Cholestan-3-ol, 2-methylene-, (3 beta, 5 alpha) derived from C. officinalis has shown activity against specific residues, such as LEU452 and ALA484, within the omicron B.1.1.529 spike protein. This suggests its potential as a targeted inhibitor against the spike protein [30]. Additionally, heneicosane from *Bontia daphnoides* L. has demonstrated a strong affinity towards bHSV type-1 thymidine kinase (TK) and HSV type-1 DNA polymerase (DP), indicating its potential as a therapeutic candidate against herpes simplex virus type-1. These findings provide valuable insights into the specific molecular interactions of these seaweed-derived compounds and highlight their potential as antiviral agents for targeted therapeutic interventions.

In summary, combining the two methods, C8, C7, C13, C11, C10, and C15 are included as part of the most potent compounds to inhibit SARS-CoV-2 and notably, C8 and C11 were rated among the other compounds by the two docking methods, AutoDock and GOLD. Beyond the results from both docking methods, the conformation, mode, and intermolecular interaction are taken carefully and analyzed for their effectiveness as inhibitors. All the compounds were bound precisely to the active site, even though some of them were not intimately bound to the cleft or the active site of each enzyme due to their size.

AutoDock is different from GOLD. Identifying the best compound from *S. polycystum* has been observed in the structural interaction between the protein complexes and each ligand (compound); however, the ranking from the two docking methods is different due to different packages and force-fields [31]. Cautious with the results from each docking method, the study found that some of the candidate compounds did not have any hydrogen bond interaction with protein, although pharmacophore has shown that the compound should have some interaction of hydrogen and bind strongly to the active site. Although different results were found using the two docking methods, the main objective was to find any candidate compounds from *S. polycystum* against SARS-CoV-2 as a drug or dietary additive by computational approach or in silico methods. These approaches have provided lots of insight and knowledge, especially the features and characteristics of SARS-CoV-2 inhibitors. The precise binding location, conformation, and free energy of each natural compound compared to the positive control from each enzyme have shown that natural compounds can compete with other well-known drugs based on their molecular interaction and binding affinity score revealed by the in-silico study. Therefore, this study could be a reference for any further drug development for SARS-CoV-2, especially from seaweed sources, and the optimization of known compounds from *S. polycystum* by in silico studies such as molecular dynamics, MM/PBSA, to obtain better insights into the ability of each compound as potential SARS-CoV-2 inhibitors.

## 5. Conclusions

In conclusion, this study has investigated natural compounds from *Sargassum polycystum* as potential antiviral agents against SARS-CoV-2. Our results suggest that the 17 natural compounds obtained from *S. polycystum* could be promising antiviral agents, particularly inhibiting the PLpro enzyme of SARS-CoV-2. While many therapeutic and nutritional benefits of *S. polycystum* have been reported, the human consumption of this abundant seaweed still needs to be popularized. With its promising beneficial effects in the prevention of SARS-CoV-2 being further elucidated, the development of seaweed-based medicine will provide additional ammunition for the growing variety of anti-SARS-CoV-2 medication.

## Figures and Tables

**Table 1:** The 17 natural compounds *Sargassum polycystum*.

No	Compounds	Smiles	PubChem ID	Area%
1	1,2-benzenedicarboxylic Acid	CC(C)CCCCCC1=C(C(=C(C=C1)C(=O)O)C(=O)O)CCCCCC(C)C	18972250	2.05
2	1,3,5-triazine-2,4-diamine, 6-chloro-n-ethyl	CCNC1=NC(=NC(=N1)N)Cl	13878	12.85
3	1,3-dioxane, 4,6-dimethyl-.	CC1CC(OCO1)C	136893	5
4	1,6-octadien-3-ol, 3,7-dimethyl	CC(=CCCC(C)(C=C)O)C	6549	2.31
5	1-decanol	CCCCCCCCCCO	154477145	2.4
6	9-octadecenoic Acid (Z)-	CCCCCCCCC=CCCCCCCCC=O	17029	3.11
7	Azulene	CC1CCC2=C(CCC(CC12)C(=C)C)C	520,826	2.07
8	Cholest-5-en-3-ol (3.Beta.)-	CC(C)CCCC(C)C1CCC2C1(CCC3C2CC=C4C3(CCC(C4)O)C)C	5997	1.51
9	Cyclohexasiloxane, Dodecamethyl-	C[Si]1(O[Si](O[Si](O[Si](O[Si](O[Si](O1)(C)C)(C)C)(C)C)(C)C)(C)C)C	10911	0.99
10	Dotriacontane	CCCCCCCCCCCCCCCCCCCCCCCCCCCCCCCC	11,008	7.9
11	Heneicosane, 11-cyclopentyl	CCCCCCCCCCC(CCCCCCCCCC)C1CCCC1	23170	2.6
12	Hexadecanoic Acid	CCCCCCCCCCCCCCCC(=O)O	985	6.66
13	Loliolide	CC1(CC(CC2(C1=CC(=O)O2)C)O)C	14334	1.05
14	Methenamine	C1N2CN3CN1CN(C2)C3	4101	6.73
15	Neophytadiene	CC(C)CCCC(C)CCCC(C)CCCC(=C)C=C	10446	3.1
16	N-formylmorpholine	C1COCCN1C=O	20417	1.59
17	Octadecanoic Acid, Methyl Ester	CCCCCCCCCCCCCCCCCC(=O)OC	8201	1.3

**Table 2:** The five most potent natural compounds against PLpro enzyme based on AutoDock software.

Protein	Molecular Docking Scores						
PLpro	Compound	Snyder 457	Cholesterol	Azulene	1,2 Benzenedicar-boxylic acid	9-octadecenoic acid (Z)	Hexadecanoic acid
	AutoDock	−10	−6.5	−6.5	−6	−5.5	−5.4
	HA	23	28	15	28	19	18
	LE	−0.43	−0.23	−0.43	−0.21	−0.29	−0.30

**Table 3:** The five most potent natural compounds against PLpro enzyme based on GOLD software.

Protein	Molecular Docking Scores						
PLpro	Compound	Synder457	Heneicosane, 11-cyclopentyl	Neophytadiene	Octadecanoic Acid, Methyl Ester	9-octadecenoic acid (Z)	Cholesterol
	GOLD	42.04	39.08	31.54	29.91	29.16	28.78

**Table 4:** The value of MDS parameters for PLpro complexes and Apo system.

Parameters	PLpro-Cholesterol	Plpro-Snyder 457	Apo System
RMSD (nm)	0.15	0.14	0.15
RMSF (nm)	0.12	0.15	0.1
Rg (nm)	2.34	2.37	2.37

**Table 5:** Calculated binding free energies of PLpro complexes.

Ligand	ΔG_vdw_	ΔG_elec._	ΔG_polar_	ΔG_surf_	ΔG_MM/PBSA_
Cholesterol	−27.58	−1.03	7.71	−3.44	−20.91
Snyder 457	−34.48	−34.41	38.23	−4.49	−30.66

## Data Availability

Not applicable.
